# Enhanced Antimicrobial and Antibiofilm Effect of New Colistin-Loaded Human Albumin Nanoparticles

**DOI:** 10.3390/antibiotics10010057

**Published:** 2021-01-08

**Authors:** Sara Scutera, Monica Argenziano, Rosaria Sparti, Federica Bessone, Gabriele Bianco, Chiara Bastiancich, Carlotta Castagnoli, Maurizio Stella, Tiziana Musso, Roberta Cavalli

**Affiliations:** 1 Department of Public Health and Pediatric Sciences, University of Turin, 10126 Turin, Italy; sara.scutera@unito.it (S.S.); rosaria.sparti@unito.it (R.S.); 2 Department of Drug Science and Technology, University of Turin, 10124 Turin, Italy; monica.argenziano@unito.it (M.A.); f.bessone@unito.it (F.B.); chiara.bastiancich@gmail.com (C.B.); roberta.cavalli@unito.it (R.C.); 3 Microbiology and Virology Unit, University Hospital Città della Salute e della Scienza di Torino, 10126 Turin, Italy; gabrielebnc87@gmail.com; 4 Institute Neurophysiopathol, INP, CNRS, Aix-Marseille University, 13005 Marseille, France; 5 Skin Bank, Department of General and Specialized Surgery, University Hospital Città della Salute e della Scienza di Torino, 10126 Turin, Italy; ccastagnoli@cittadellasalute.to.it; 6 Burn Center, CTO Hospital, Città della Salute e della Scienza di Torino, 10126 Turin, Italy; stella.maurizio@libero.it

**Keywords:** colistin, human albumin, nanoparticles, MDR strains, biofilm

## Abstract

Multidrug-resistant (MDR) Gram-negative bacteria (GNB), such as *Acinetobacter* and *Klebsiella*, are responsible for severe hospital-acquired infections. Colistin, despite its toxicity and low tissue penetration, is considered the last resort antibiotic against these microorganisms. Of concern, the use of Colistin has recently been compromised by the emergence of Colistin resistance. Herein, we developed a new formulation consisting of multifunctional chitosan-coated human albumin nanoparticles for the delivery of Colistin (Col/haNPs). Col/haNPs were in vitro characterized for encapsulation efficiency, drug release, stability and cytotoxicity and were evaluated for antibacterial activity against MDR GNB (*Acinetobacter baumannii* and *Klebsiella pneumoniae*). Col/haNPs showed sizes lower than 200 nm, high encapsulation efficiency (98.65%) and prolonged in vitro release of Colistin. The safety of the nanoformulation was demonstrated by a negligible cytotoxicity on human fibroblasts and hemolytic activity. Col/haNPs evidenced a high antibacterial effect with a significant decrease in MIC values compared to free Colistin, in particular against Col-resistant strains with a pronounced decline of bacterial growth over time. Moreover, Col/haNPs exhibited an inhibitory effect on biofilm formation that was 4 and 60 fold higher compared to free Colistin, respectively for Colistin susceptible and resistant *A. baumannii*. Our findings suggest that Col/haNPs could represent a promising Colistin nanocarrier with high antimicrobial activity on MDR GNB.

## 1. Introduction

The antimicrobial resistance phenomenon has long been an important sanitary emergency. In the last two decades nosocomial Gram-negative bacteria (GNB), such as the Enterobacteriaceae *Klebsiella pneumoniae* and *Escherichia coli*, *Acinetobacter baumannii* and *Pseudomonas aeruginosa*, have become multidrug-resistant (MDR), extensively drug-resistant (XDR), and in some cases also pan-drug-resistant (PDR) [1]. The resulting difficulties in treating GNB infections made it necessary to resort to “last choice” antibiotics such as Colistin (Col), a polymyxin family member, first used between 1950s and 1960s and then abandoned due to its nephro- and neuro-toxicity. Col is available for clinical use in two forms: Col sulphate, used topically to treat skin infections or orally for enteral decontamination, and the Col methanesulfonate (ColM), a prodrug administered parenterally or by inhalation to manage systemic and pulmonary infections respectively. Apart from toxicity, Col has the disadvantage to poorly penetrate different sites (pleura, bones, lung parenchyma and CSF) and to induce bacterial regrowth, even at concentrations higher than the MIC [2,3].

Col exerts its antimicrobial action by binding to the negative charge of LPS of the outer membrane of GNB that leads to an increase in membrane permeability and consequently to bacterial lysis. Other mechanisms and targets of Col action have been also proposed, such as the production of the reactive oxygen species and the inhibition of respiratory enzymes [4,5]. Unfortunately, several mechanisms of resistance to Col have been recently discovered. Col resistance is mostly mediated by chromosomal mutations of genes involved in LPS biosynthesis or in glucose transport pathways. However, horizontally acquired resistance mechanism through the plasmid-mediated mcr-1 gene has been recently demonstrated. Moreover, some bacteria such as *Serratia*, *Proteus* and *Burkholderia* spp. are intrinsically resistant to Col by LPS modification [6,7].

Given the increasing prevalence of Col resistance, the search for new formulations of this old antibiotic is of great interest. Antibiotic encapsulation can enhance its effect against bacteria and overcome antibiotic resistance. Several nanocarriers, such as liposomes, micelles, solid lipid nanoparticles and polymeric nanoparticles, have been proposed for antibiotic delivery to increase drug concentration and penetration at the infection sites. In addition, the nanoformulation can overcome limitations associated with antibiotic administration, such as poor drug solubility, stability and low permeation through biological barriers [8,9,10,11]. Moreover, a nanotechnological approach is among the most promising strategies to counteract resistant bacterial infections, by affecting the antibiotic-pathogen interactions [12]. As an example, meropenem-loaded chitosan nanoparticles have demonstrated great potential for the management of antimicrobial resistance, showing higher in vitro antibacterial activity against clinical isolates of multidrug-resistant *E. coli*, *K. pneumoniae* and MRSA, in comparison to free drugs. Furthermore, a prolonged bactericidal activity was demonstrated in an in vivo model of systemic infection of meropenem-sensitive *K. pneumoniae* [13]. The antibacterial activity of ciprofloxacin against *P. aeruginosa, K. pneumoniae* and *S. pneumoniae* species was enhanced by its incorporation in chitosan nanomicelles [14].

The goal of the study was to design and develop a new formulation of multifunctional chitosan-coated human albumin nanoparticles for the delivery of Col (Col/haNPs). This new antibiotic delivery system was evaluated for antimicrobial and antibiofilm effect on resistance and sensible to Col GNB.

## 2. Results

### 2.1. Characterization of Col/haNPs

The haNPs showed sizes of about 175 nm and positive surface charge, due to the chitosan coating. Table 1 reports the physico-chemical characteristic of haNPs either blank or Col loaded.

The incorporation of Col into the haNPs did not modify the zeta potential and average diameter values of haNPs. SEM analysis confirmed the spherical morphology of Col/haNPs and their nanometric sizes (data not shown). Col was incorporated in haNPs to a good extent, showing an encapsulation efficiency of 98.65 ± 0.15%. The physical stability of Col/haNPs stored at 4 °C was evaluated up to 6 months. No significant modifications in their physico-chemical characteristics or aggregation phenomena were observed (Figure S1). The content of Col in the haNPs did not change over time. Indeed, the 99.85 ± 0.02 % of the Col initial concentration was found after 6 months.

**Figure 1:**
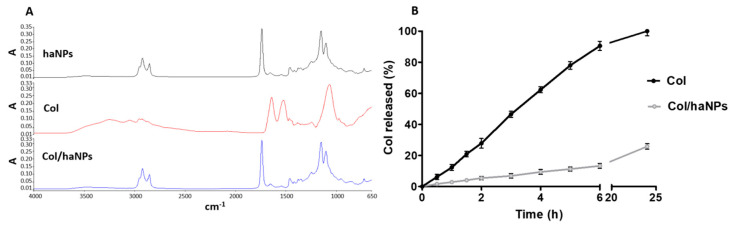
(**A**) FTIR spectra of blank haNPs, Col and Col/haNPs. (**B**) In vitro release kinetics of Col from Col/haNPs.

FTIR analysis of Col/haNPs did not present the peaks related to the drug, showing the incorporation of Col in the haNPs (Figure 1A). Indeed, the characteristic bands of Col at 1645 and 1538 cm^−1^ (Amide I and Amide II bands) and the band at 1099 cm^−1^, from antisymmetric stretching related to the sulphate counter ion, were not detected in the Col/haNPs spectrum [15]. This result indicated the encapsulation of Col in nanoreservoirs within the albumin matrix.

The in vitro release kinetics of Col from Col/haNPs is shown in Figure 1B. An HPLC analysis was carried out to determine the amount of Col released over time from Col/haNPs.

A prolonged in vitro release profile was observed, reaching about the 26% of Col released from the haNPs after 24 h at pH 7.4. In these experimental conditions, the chitosan aminogroups of the haNP coating are not protonated, favoring the formation of a thick layer. The absence of a burst effect suggested that Col is incorporated in the albumin matrix and not only adsorbed on the NP surface. The in vitro release results were fitted to mathematical kinetic models (i.e., zero-order kinetic model, first-order kinetic model, simplified Higuchi model and Korsmeyer-Peppas model) to evaluate the possible mechanisms of drug release from the haNPs. The rate constants and correlation coefficients, calculated by applying a linear regression fit to each graph, are reported in Table 2.

The in vitro Col release profile from Col/haNPs showed the best correlation with the simplified Higuchi model (R^2^ 0.98), indicating that Col is mainly released by means of a diffusion-controlled mechanism in a sustained manner.

### 2.2. Antimicrobial Capacity of Col/haNPs

#### 2.2.1. Minimum Inhibitory Concentration (MIC) of Col/haNPs

The antibacterial activity was determined against *Acinetobacter baumannii* ATCC19606 and clinical strains obtained from the Microbiology and Virology Unit, AOU Città della Salute e della Scienza di Torino. The susceptibility profile to conventional drugs of the different strains used is presented in Table 3. All clinical strains show an MDR profile.

The susceptibility profile of free Col and Col/haNPs has been also determined using the broth microdilution method. Col S strains are susceptible to free Col according to the breakpoint defined by European Committee on Antimicrobial Susceptibility Testing (EUCAST) (>2 μg/mL), while Col-resistant *Acinetobacter baumannii* and *Klebsiella* strains show MIC values ranging from 20 to >40 μg/mL. Col/haNPs exhibited a higher antibacterial activity when compared to free Col as indicated by the significant decrease in the MIC values. A two-fold reduction (from 0.31 to 0.156 Col vs. NPs) was observed for *the Acinetobacter baumannii* ATCC 19606 and *Acinetobacter baumannii* Col S. Interestingly, we observed a significant reduction in MIC values for *Acinetobacter baumannii* Col R strains (>40 to 2.5/1.25 μg/mL Col vs. Col/haNPs) and for *Klebsiella pneuomoniae* KPC resistant to Col strains (20/40 to 2.5/0.62 μg/mL Col vs. Col/haNPs). An equivalent amount of empty haNPs to one of the Col/haNPs did not show any antibacterial effect. Similarly, no effect was observed for free albumin (up to 4.28 mg/mL) (Table 4).

#### 2.2.2. Effect of Col/haNPs on Bacterial Growth

**Figure 2:**
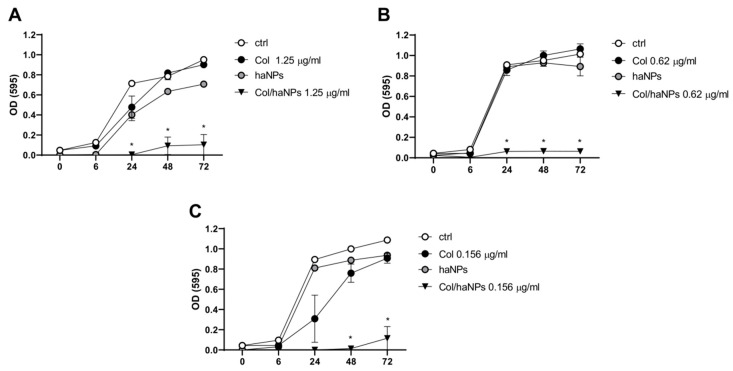
Microbial growth kinetics of *A. baumannii* and *K. pneumoniae* KPC in the presence of Col, haNPs and Col/haNPs. (**A**) Colistin-resistant *A. baumannii* (Col R1). (**B**) Colistin-resistant *K. pneumoniae* KPC2. (**C**) Colistin-susceptible *A. baumannii* (Col S). The treatment concentration corresponds to the MIC of Col/haNPs. Untreated bacteria were used as control. The microbial growth was measured as optical density (OD 595). Results are the mean values ± SEM of three independent experiments. * *p* < 0.05 indicates statistical significance as compared to free Col by Student’s *t*-test.

The growth curves of both Col resistant (*A. baumannii* Col R1 and *K. pneumoniae* KPC) and susceptible (*A. baumannii* Col S) strains were determined in the presence of Col/haNPs at their MIC values, haNPs, and equal amount of free Col. Growth rates were determined by measuring OD at 595 nm at different time points (6, 24, 48 and 72 h). After a log phase of about 6 h, untreated control cultures showed a sharp increase in OD and a stationary phase was reached at times over 24 h (followed by a slight increase up to 72 h). A similar pattern of growth was observed for *A. baumannii* Col R1 and *K. pneumoniae* KPC resistant to Col in the presence of free Col. Addition of Col/haNPs to both *A. baumannii* Col R1 and *K. pneumoniae* KPC was followed by a pronounced decline of the OD (Figure 2A,B). *A. baumannii* Col S showed a slightly reduced growth in the presence of free Col (mainly at 24 h), compared to untreated bacteria, consistent with a possible effect of a sub MIC concentration (0.156 vs. 0.31 μg/mL). Instead, the addition of Col/haNPs drastically limited bacterial growth up to 72 h (fully inhibits up to 48 h) (Figure 2C). haNPs did not have significant antibacterial activity.

#### 2.2.3. Antibiofilm Effect

**Figure 3:**
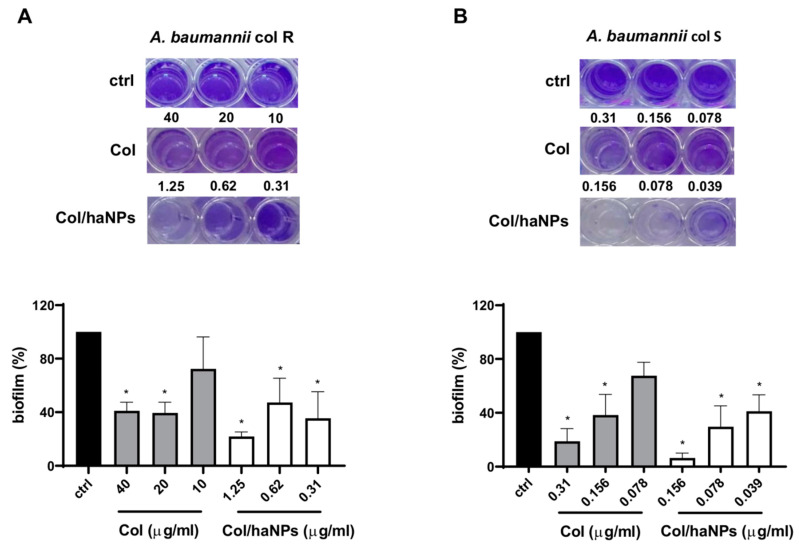
Antibiofilm activity of Col and Col/haNPs on *A. baumannii* Col R1 (**A**) and *A.baumannii* Col S (**B**). Bacteria were treated at concentrations of MIC, ½ MIC and ¼ MIC. Biofilm formation of bacteria untreated was used as control and set as 100%. Results are the mean values ± SEM of three independent experiments. * *p* < 0.05 indicates statistical significance as compared to control by Student’s *t*-test.

Given the importance of biofilm in bacterial infections, we evaluated the capability of Col/haNPs to counteract biofilm formation. To this purpose, *A. baumannii* Col R1 and *A. baumannii* Col S were treated with different concentrations of Col/haNPs and Col free (MIC, ½ MIC and ¼ MIC) and evaluated for biofilm formation after 24 h. Col/haNPs displayed potent biofilm inhibition against both Colistin susceptible and resistant *A. baumannii* strains and significantly reduced the biofilm also at ¼ MIC (0.31 and 0.039 μg/mL for *A. baumannii* Col R1 and S, respectively) (Figure 3). By contrast, Col free is able to reduce biofilm of *A. baumannii* Col R1 only at high concentrations (20 and 40 μg/mL) while it can affect biofilm formation of *A. baumannii* Col S up to ½ MIC (0.156 μg/mL). The inhibitor effect obtained with Col/haNPs is about 4 fold higher compared to that of free Colistin for *A. baumannii* Colistin-susceptible and about 60 fold higher for the resistant strain.

### 2.3. Cytocompatibility of Col/haNPs

#### 2.3.1. In Vitro Hemolytic Activity

**Figure 4:**
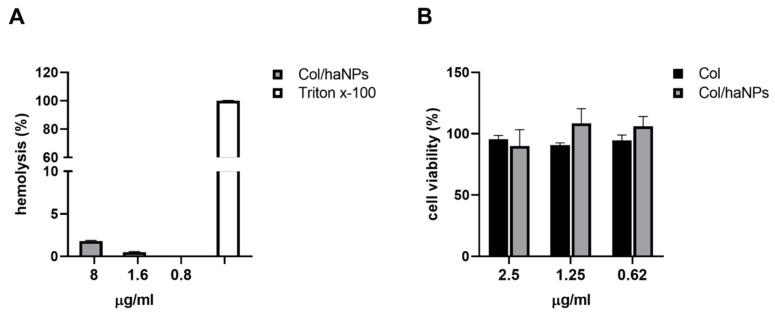
Col/haNPs biocompatibility. (**A**) Percentage of hemolysis induced by Col/haNPs at different concentrations. Triton x-100 was used as a positive control. (**B**) Graphical representation of the cytotoxic effect of Col/haNPs in comparison with Col free used at different concentrations (from the highest MIC of resistant strains) on HFF fibroblasts after treatment for 24 h. Untreated cells were used as control and set as 100%. Results are the mean values ± SEM of three independent experiments.

Negligible hemolytic activity (≤2%) of Col/haNPs was observed even at concentrations higher than MIC (Figure 4A), confirming the biocompatibility of the nanoparticles. Free Col had no hemolytic effect as previously reported (data not shown) [16].

#### 2.3.2. In Vitro Cytotoxicity Effect

The cytocompatibility of Col/haNPs and free Col was evaluated using the human cell line HFF. Cell viability test demonstrated that HFFs exposed to Col/haNPs, used at different concentrations (starting from the highest MIC concentration to decrease), presented a percentage of viable cells comparable to that of untreated or Col-treated cells, indicating that at the usage concentrations nanocarriers are not toxic and biocompatible (Figure 4B).

## 3. Discussion

Severe healthcare infections associated with the Gram-negative bacteria MDR *A. baumannii* and *Enterobacteriaceae*, have significantly increased in the last decades [17]. One of the major virulence factors of these bacteria is their ability to form a biofilm that prevents antibiotic activity as well as host immune response. Moreover, to make the treatment even more difficult, they have acquired a new resistance mechanism to last resort antibiotics such as polymyxin [2].

In the last few years new strategies have been focused on old antibiotic combinations, phage therapy, usage of antimicrobial peptides and on the development of nanomedicine-based forms of existing antimicrobial drugs [1,18,19,20]. Recently, several delivery systems for Col have been developed to improve bioavailability and reduce its toxicity, to be used both for topic and for inhalation and parental administration. For example, lipid-based nanostructures encapsulating Col were proposed by several authors [21,22,23,24,25] and different chitosan-based nanocarriers have been shown to improve Col antimicrobial activity [26,27]. Moreover, recently Ran HH et al. [28] developed Colistin-loaded polydopamine nanospheres decorated with silver nanodots with improved antimicrobial and antibiofilm activity. In this contest, protein-based polymers such as albumin have been considered as drug carriers due to their non-toxicity, biocompatibility, biodegradability and poor immunogenic properties [29]. Albumin NPs have been largely proposed for the delivery of anticancer drugs and approved in the first-line treatment of metastatic breast cancer [30,31].

In the present study we proposed a new formulation comprising chitosan-coated human albumin nanoparticles loaded with Col that demonstrated cytocompatibility and a high antimicrobial and antibiofilm efficacy against MDR strains of *A. baumannii* and *K. pneuomaniae* KPC.

The selection of human albumin for the formulation considered the several advantages associated with this attractive macromolecular carrier, such as the presence of reactive functional groups in its structure (thiol, amino and carboxylic groups) that enables the binding of compounds with different chemical features. A tuned preparation process exploiting a double emulsion (W/O/W) to obtain the encapsulation of the antibiotic within the albumin matrix was developed. The method allowed the formation of loaded haNPs in mild conditions and without the use of any solvents. In addition, the Col incorporation in the ha/NPs did not affect the physico-chemical properties and stability of haNPs. The haNPs were then coated with a positively charged chitosan layer, exploiting the electrostatic interaction with the negatively charged albumin NP surface. Indeed, the well-defined structure of the protein-containing charged amino acids favored the electrostatic adsorption of positively charged molecules [32]. The complexation of proteins and polysaccharides via non covalent interactions, such as hydrophobic and electrostatic ones, has been previously evaluated for the formulation of nanoparticles [33,34]. Even if recently antibacterial properties of albumin have been also hypothesized [35,36,37], albumin nanoparticles for antibiotic delivery have not been deeply investigated and to date no such formulations loaded with Colistin have been developed.

Chitosan, a natural polycationic polysaccharide, was chosen as a coating biomaterial thanks to its excellent biocompatibility, biodegradability, mucoadhesive and antibacterial properties [38]. Interestingly, the chitosan mucoadhesion capability might be exploited to increase the residence time of Col/haNPs in the target site, favoring a prolonged drug release at the infection site and the absorption of the antibiotic. Consequently, these features might allow the decrease of dose and administration frequency. In addition, the combination of chitosan with Col might result in an antibacterial synergistic effect, as previously reported for other antibiotic molecules [39,40].

We demonstrated a remarkable antimicrobial activity of our Col/haNPs on different strains of *A. baumannii* and *K. pneumoniae*, able to inhibit the bacterial growth with MIC values lower of at least 2 and 8 fold compared to free Col in susceptible and resistant bacteria, respectively. The effect persisted for long time, confirming the constant and sustained release of Col from the nanoparticles observed in vitro. Considering that drug release in vitro from nanoparticles was about 26% after 24 h, the real antibacterial concentrations would be even lower, demonstrating even more the effectiveness of the NP formulation.

We assume that our positive charged nanoparticles tend to be adsorbed and accumulated on the negatively charged bacterial surface by electrostatic interactions in both MDR and susceptible Gram negative strains, causing membrane destabilization and cell death. We cannot exclude that other mechanisms, such as induction of oxidative stress, increased uptake or decreased efflux of the encapsulated drug, are involved in the Col/haNPs effect overcoming resistance defenses [41]. Further studies are needed to better clarify the antibacterial mode of action of this formulation and the possible correlation with specific Col resistance mechanisms.

The prevalence of these bacteria in nosocomial infections has been associated with the capacity of biofilm developing [42,43,44], causing an advantage for the microbe and the risk for persistent infections. Different studies indicated that Col could reduce biofilm formation of *A. baumannii* and *K. pneumoniae* at its sub-MIC [43], while others reported that biofilm was not affected or increased by Col treatment at sub-MICs [45]. We found that both biofilm formation by Col susceptible and resistant *A. baumannii* was significantly inhibited using sub-MIC doses of Col/haNPs (until ¼ MIC), while free Col was able to reduce biofilm until ½ MIC. Moreover, in Col-resistant strains, Col/haNPs brought significant changes in biofilm formation, using up to 40 times lower doses than free Col. Compared to results obtained by Pastor M et al. [46] using sodium Colistin methate-loaded lipid nanocarries and by Sans-Serramitjiana E et al. [23] using nanoencapsulated Colistin sulfate that demonstrated an identical activity of free and loaded Colistin against biofilm prevention of *P. aeruginosa*, we noticed a great effect of our albumin nanoparticles to prevent biofilm formation of *A. baumannii*. Recently, an effect of PEG-cHSA (cationic human serum albumin) was observed only using MIC and supra-MIC concentrations to prevent *P. aeruginosa* biofilms [35]. The biofilm-modulating action of MIC and sub-MICs depends on many factors: type of antimicrobial, mode of action, bacterial strain and susceptibility or resistance of the strain. Moreover, a not-linear dose-dependent effect on biofilm modulation has been reported [47,48]. 

The struggle to bring new antimicrobial agents to market, although a disincentive by pharmaceutical industries, has become a priority. All this has brought to the fore the words of the Nobel pharmacologist Sir James Black: “The most fruitful basis for the discovery of a new drug is to start with an old drug” [49].

Here, we characterized and demonstrated that Col/haNPs are high efficient to counteract *A. baumannii* and *K. pneumoniae* KPC growth and biofilm formation. This study deserves further attention owing to its potential application to fight bacterial infections caused by Col-resistant bacteria.

## 4. Materials and Methods

### 4.1. Preparation and Characterization of Chitosan-Coated Albumin Nanoparticles for the Delivery of Col

#### 4.1.1. Preparation of Blank and Colistin-Loaded Chitosan-Coated Albumin Nanoparticles

Blank and Colistin-loaded albumin nanoparticles (haNPs) were prepared by a purposely tuned double-emulsion method, as reported in the patent (Italian Patent N° 102020000022984, University of Turin). Briefly, a weighted amount of colistin was dissolved in an aqueous solution of Span83™ (1% *w*/*v*). A first emulsion was obtained adding this Col solution to 2 mL of Miglyol^®^ and sonicating for 1 min using an Ultrasonic Generator (20 K, 500 W; Hainrtec, type HNG-20500-SP). Then, 200 µL of the first emulsion was dropwise added to 3 mL of an albumin solution (10 mg/mL), prepared dissolving Human Albumin in a TRIS buffer (25 mM, pH 8.0), containing Tween^®^ 80 (2.5% *w*/*v*) as surfactant. The system was sonicated for 1 min using an Ultrasonic Generator to obtain the haNPs.

Then, the formed haNPs were coated with a chitosan layer. For this purpose, an aqueous solution of chitosan (2.7% *w*/*v*, pH 4.5) was dropwise added under magnetic stirring to the preformed albumin nanoparticles. Finally, a dialysis step against saline solution (NaCl 0.9% *w*/*v*) was performed to eliminate the unloaded Col from the Col/haNP nanosuspension. Blank haNPs were formulated in the absence of Col using the same procedure.

#### 4.1.2. Characterization of Col-Loaded Chitosan-Coated Albumin Nanoparticles

The chitosan-coated albumin nanoparticles, either blank or Colistin loaded, were in vitro characterized measuring their physico-chemical parameters.

The average diameter, polydispersity index and zeta potential of albumin NPs were determined by Dynamic Light Scattering (DLS) using a 90 Plus Instrument (Brookhaven, NY, USA). The analyses were performed at a scattering angle of 90° and at 25 °C, using samples diluted in water (1:30 *v*/*v*). The morphology of the haNPs was observed by Scanning Electron Microscopy (SEM) analysis using a TESCAN S9000G instrument. Fourier transformed infrared (FTIR) spectra of the Col, Col/haNPs and blank haNPs were collected using a Perkin Elmer Spectrum 100 FT-IR in the region 4000^−1^–650^−1^. Data acquisition was carried out using spectrum software version 10.03.05 Perkin Elmer Corporation.

#### 4.1.3. HPLC Quantitative Determination of Colistin

The quantitative determination of Col was carried out using an HPLC system consisting of a PerkinElmer PUMP 250B, equipped with a Flexar UV/Vis LC spectrophotometer detector (PerkinElmer, Waltham, MA, USA). A reversed phase Agilent TC C18 column (250 mm × 4.6 mm, pore size 5 μm; Agilent Technologies, Santa Clara, CA, USA) was used. The mobile phase consisting of a mixture of acetonitrile and 1.2 mM sodium sulfate (70:30 *v*/*v*) was pumped through the column at 1 mL/min flow rate. The eluent was monitored using an UV detector set at 210 nm. The Col concentration was calculated using the external standard method from a standard calibration curve, linear over a 0–25 μg/mL concentration range (regression coefficient of 0.999).

#### 4.1.4. Determination of Encapsulation Efficiency

The encapsulation efficiency of Col/haNPs were determined on freeze-dried samples. A weighted amount of freeze-dried Col/haNPs were dispersed in 10 mL of water and sonicated for 30 min. After centrifugation (10 min, 15000 rpm) the supernatant was analyzed by HPLC to determine the amount of Colistin incorporated in the albumin NPs.

The encapsulation efficiency was calculated according to the following equation:

[amount of Colistin in NPs/total amount of Colistin] × 100.

#### 4.1.5. In Vitro Release Studies

The in vitro release kinetics of Col from Col/haNPs was investigated using a multicompartment rotating cell. A dialysis membrane (Spectra/Por cellulose membrane, cutoff 14 KDa) was used to separate the donor compartment, containing 1 mL of Col/haNPs or Colistin solution, from the receiving phase, consisting of 1 mL of phosphate buffered saline (PBS) at pH 7.4. At fixed times the receiving phase was withdrawn and replaced with the same amount of fresh PBS, to maintain the sink conditions. The withdrawn samples were analyzed by HPLC analysis, to determine the amount of Colistin released over time.

Mechanisms of drug release were investigated by fitting the in vitro experimental results to four mathematical kinetic models (i.e., zero-order kinetic model, first-order kinetic model, simplified Higuchi model, and Korsmeyer–Peppas model). The cumulative % of drug release vs. time was plotted for the zero-order kinetic model and the log cumulative % of drug remaining vs. time for first-order kinetic model. The Higuchi model was analyzed by plotting cumulative % drug release vs. square root of time, while the Korsmeyer–Peppas model was plotted by log cumulative % drug release vs. log time. The correlation coefficients were calculated by applying a linear regression fit to each graph [50].

#### 4.1.6. In Vitro Stability Studies

The physical stability of Col/haNPs stored at 4 °C was evaluated determining their physico-chemical parameters and Colistin content over time.

#### 4.1.7. Hemolytic Activity Determination

The hemolytic activity of Col/haNPs was evaluated on rat blood diluted with PBS pH 7.4 (1:10 *v*/*v*). The samples were incubated with 1 mL of blood at different ratios (1:10, 1:25, 1:50, 1:100, 1:200, 1:400 *v*/*v*) at 37 °C for 90 min. After incubation, the samples were centrifuged at 2000 rpm for 10 min to separate the plasma. The amount of hemoglobin released in the supernatant due to hemolysis was measured spectrophotometrically at 543 nm (Du 730 spectrophotometer, Beckman). The hemolytic activity was calculated with reference to complete hemolyzed samples (induced by the addition of Triton X-100 1% *w*/*v* to the blood, used as positive control) and negative control (NaCl 0.9% *w*/*v*).

#### 4.1.8. Cytotoxicity Assay

The Col/haNPs biocompatibility was evaluated using HFF (human foreskin fibroblasts) cell line. Cells were cultured overnight at 0.5 × 10^6^ cells/mL in 6-well-plate at 37 °C and 5% CO_2_ in Dulbecco’s modified Eagle’s medium (DMEM) supplemented with 10% Fetal Bovine Serum (FBS), 2 mM glutamine and 1% antibiotics (Gibco, Thermo Fisher Scientific, Waltham, MA, USA). After overnight incubation cells were treated for 24 h with Col free or Col/haNPs at different concentrations. The viability of fibroblasts was measured according to the 3-(4,5-dimethylthiazol-2-yl)-2,5-diphenyltetrazoliumbromide (MTT) assay protocol. Each experimental condition was performed in triplicate and OD was measured at 570 nm using a microplate reader (VICTOR3TM, PerkinElmer, MA, USA).

### 4.2. Microbiological Experiments

#### 4.2.1. Microorganisms

The following bacterial strains were used in the study: *Acinetobacter baumannii ATCC19606*, one *Acinetobacter baumannii* clinical isolate Colistin-susceptible (Col S), three *Acinetobacter baumannii* isolates Colistin-resistant (Col R1, R2 and R3) and two *Klebsiella pneumoniae* carbapenem—resistant isolates (KPC1 and KPC2).

#### 4.2.2. Susceptibility Testing

Antimicrobial susceptibility was tested by MicroScan WalkAway 96 SI instrument using the NMDR panel (Beckman Coulter srl, Milan, Italy), according to the manufacturer’s instructions. The minimum inhibitory concentration (MIC) of Colistin, free albumin, albumin nanoparticles (haNPs) or Colistin-loaded albumin nanoparticles (Col/haNPs) for the abovementioned strains was determined according to EUCAST recommendations using a broth microdilution method in ninety six well microtitre plates containing 200 μL Muller Hinton (MH) broth. Free Colistin and Col/haNPs were diluted in serial twofold dilutions (final concentrations between 80 to 0.039 μg/mL for free Colistin and 40 to 0.019 μg/mL for the nanoparticles).

The log phase bacterial suspensions were diluted with saline and inoculated in the wells to give a final inoculum concentration of 1 × 10^5^ CFU/mL. The microtiter plates were incubated at 37 °C and were scored visually for growth or no growth after 24 h. The lowest concentration of compound tested, inhibiting growth was reported as the minimum inhibitory concentration (MIC). Resistance breakpoint was set as >2 based on Eucast guidelines and quality control was performed with a susceptible QC strain (*Acinetobacter baumannii ATCC19606*).

#### 4.2.3. Assessment of Biofilm Formation

We selected two *Acinetobacter baumannii* biofilm-forming isolates, one Colistin-susceptible and one Colistin resistant. The quantification of planktonic and biofilm cells was performed in 96-well-plates. The bacteria were inoculated at a density of approximately 1 × 10^5^ CFU/mL in LB broth with antibiotics present at their MICs and sub-MICs (1/4, 1/2); 0 MIC represents untreated bacteria. They were incubated for 24 h at 37 °C. After incubation, the supernatant from each well was transferred to a corresponding well in a new plate and OD was measured at 595 nm using a microplate reader (VICTOR3TM, PerkinElmer, MA, USA). This was used to define the number of planktonic cells. Any biofilm cells remaining in each well were washed three times with sterile distilled water, followed by staining with 1% crystal violet solution for 15 min. The cells were then washed three times with sterile distilled water and air-dried for an hour. Stained biofilm cells were de-stained using 95% ethanol; the OD was measured again at 595 nm. This was used to define the number of biofilm cells. The results were expressed as a percentage of control determined by the equation: [(A/A_0_) × 100], where A_0_ and A are absorbance, i.e., OD, at 0 MIC and 1/4, 1/2 MIC, MIC of antibiotics, respectively.

#### 4.2.4. Microbial Growth Kinetics Assay

Colistin-resistant *A. baumannii* R1 and *K. pneumoniae* KPC2 and Colistin-susceptible *A. baumannii S* were used to compare the effect of free, nanoencapsulated Colistin and blank nanoparticles on bacterial growth.

Bacteria were incubated for 0 h, 6 h, 24 h and 72 h in 96-well-plate in Muller Hinton (MH) broth at 37 °C at a starting concentration of 1 × 10^5^ CFU/mL with MIC, ½ MIC and ¼ MIC antimicrobial concentrations. At the end of the exposure time the growth was measured as optical density (595 nm) using a microplate reader (VICTOR3TM, PerkinElmer, MA, USA).

#### 4.2.5. Statistical Analysis

Statistical significance was determined by using non-parametric Student’s *t*-test. Results were analyzed with GraphPad PRISM 8.0 software.

## 5. Patents

Italian Patent N° 102020000022984 (University of Turin, Italy): “Composizione comprendente nanoparticelle di albumina incapsulanti antibiotici”.

## Figures and Tables

**Table 1:** Physico-chemical parameters of blank and Col loaded haNPs.

Sample	Average Diameter ± SD (nm)	PDI	Zeta Potential ± SD (mV)	pH
blank haNPs	174.7 ± 2.1	0.20	29.76 ± 1.5	7.52
Col/haNPs	176.1 ± 0.9	0.12	28.05 ± 2.1	7.46

**Table 2:** Rate constants and correlation coefficients obtained from modeling Col release kinetics from Col loaded haNPs through the following mathematical kinetic models: zero-order kinetic model, first-order kinetic model, simplified Higuchi model and Korsmeyer–Peppas model.

Kinetics Model	Col/haNPs	
	K	R^2^
Zero-order	0.9279	0.8708
First-order	0.0047	0.8982
Simplified Higuchi	5.2381	0.9774
Korsmeyer-Peppas	0.7189	0.9734

**Table 3:** Antibiotic susceptibility profiles of strains used in the study.

Antibiotics		MDR Clinical Strains					
	*A. baumannii* ATCC 19606	*A. baumannii* Col S	*A. baumannii* Col R1	*A. baumannii* Col R2	*A. baumannii* Col R3	*K. pneumoniae* KPC 1	*K. pneumoniae* KPC 2
PIP/TAZO	NT	NT	NT	NT	NT	>16 (R)	>16 (R)
MEM	1 (S)	>8 (R)	>8 (R)	>8 (R)	8 (S)	>8 (R)	>8 (R)
IMP	≤1 (S)	>8 (R)	>8 (R)	>8 (R)	>8 (R)	>8 (R)	>8 (R)
ERT	NT	NT	NT	NT	NT	>1 (R)	>1 (R)
CAZ	NT	NT	NT	NT	NT	>8 (R)	>8 (R)
CTX	NT	NT	NT	NT	NT	>16 (R)	>16 (R)
CEFE	NT	NT	NT	NT	NT	>8 (R)	>8 (R)
GENTA	>4 (R)	>4 (R)	>4 (R)	>4 (R)	>4 (R)	4 (R)	4 (R)
AMIKA	NT	NT	>16 (R)	NT	NT	>16 (R)	>16 (R)
FOSFO	NT	NT	NT	NT	NT	>32 (R)	>32 (R)
SXT	>4/76 (R)	>4/76 (R)	>4/76 (R)	>4/76 (R)	>4/76 (R)	>4/76 (R)	≤2/38 (S)
CIPRO	1(S)	>1 (R)	>1 (R)	>1 (R)	>1 (R)	>1 (R)	NT
LEVO	≤0.5 (S)	>1 (R)	>1 (R)	>1 (R)	>1 (R)	>1 (R)	>1 (R)
CAZ/AVI	NT	NT	NT	NT	NT	≤2 (S)	≤2 (S)
COL	≤2 (S)	≤2 (S)	>4 (R)	>4 (R)	>4 (R)	>4 (R)	>4 (R)

MIC values (μg/mL); NT: not tested; PIP/TAZO: Piperacillin/tazobactam; MEM: Meropenem; IMP: Imipenem; ERT: Ertapenem; CAZ: Ceftazidime; CTX: Cefotaxime; CEFE: Cefepime; GENTA: Gentamicin; AMIKA: Amikacin; FOSFO: Fosfomycin; SXT: Trimethoprim/Sulfamethoxazole; CIPRO: Ciprofloxacin; LEVO: Levofloxacin; CAZ/AVI: Ceftazidime-Avibactam; COL: colistin.

**Table 4:** Col and Col/haNPs MIC of bacteria strains analyzed.

Pathogenic Bacteria	Col MIC (μg/mL)	Col/haNPs MIC (μg/mL)
*Acinetobacter baumannii* ATCC19606	0.31	0.156
*Acinetobacter baumannii* Col S	0.31	0.156
*Acinetobacter baumannii* Col R 1	>40	1.25
*Acinetobacter baumannii* Col R 2	>40	1.25
*Acinetobacter baumannii* Col R 3	>40	2.5
*Klebsiella pneumoniae* KPC 1	20	2.5
*Klebsiella pneumoniae* KPC 2	40	0.62

## Data Availability

The data presented in this study are available on request from the corresponding author.

## Supplementary Materials

The following are available online at https://www.mdpi.com/2079-6382/10/1/57/s1, Figure S1: Average diameter and Zeta potential values of Col loaded haNPs.
