# Supplementary material for: Enhanced Antimicrobial and Antibiofilm Effect of New Colistin-Loaded Human Albumin Nanoparticles
Source: Antibiotics (Basel). 2021 Jan 8;10(1):57. doi: 10.3390/antibiotics10010057 (PMC7827731; doi:10.3390/antibiotics10010057)
Supplement: Supplementary file 1 [file antibiotics-10-00057-s001.pdf]

Supplementary Materials

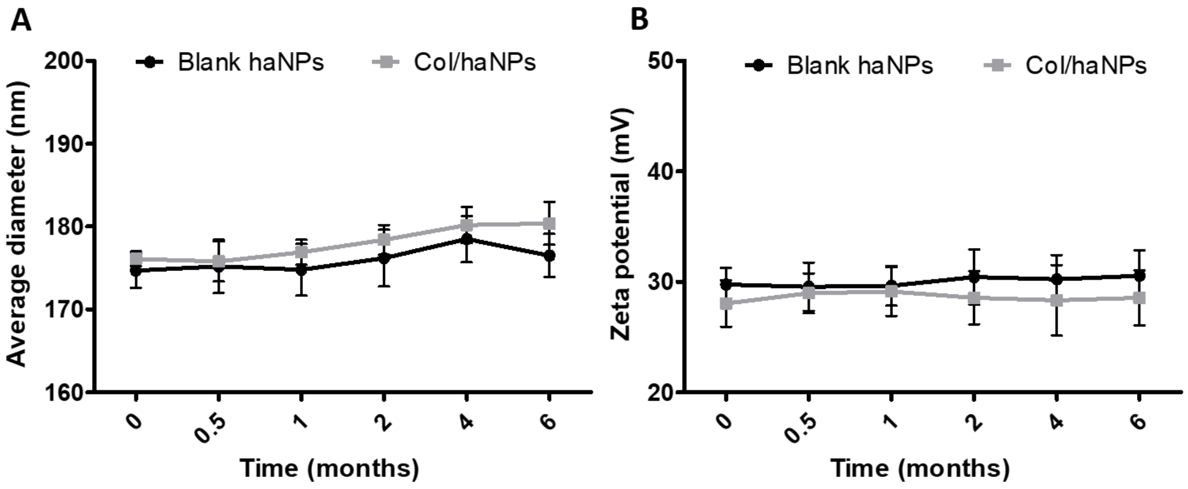

Figure S1. Average diameter (A) and Zeta potential (B) values of blank and Col loaded haNPs over time. Each point represents the mean ± SD of 3 experiments.
